# Static and Fatigue Tests on Cementitious Cantilever Beams Using Nanoindenter

**DOI:** 10.3390/mi9120630

**Published:** 2018-11-28

**Authors:** Yidong Gan, Hongzhi Zhang, Branko Šavija, Erik Schlangen, Klaas van Breugel

**Affiliations:** Microlab, Faculty of Civil Engineering and Geosciences, Delft University of Technology, 2628 CN Delft, The Netherlands; y.gan@tudelft.nl (Y.G.); B.Savija@tudelft.nl (B.Š.); Erik.Schlangen@tudelft.nl (E.S.); K.vanBreugel@tudelft.nl (K.v.B.)

**Keywords:** cement paste, miniaturized cantilever beam, micromechanics, fatigue, nanoindenter

## Abstract

Cement paste is the main binding component in concrete and thus its fundamental properties are of great significance for understanding the fracture behaviour as well as the ageing process of concrete. One major aim of this paper is to characterize the micromechanical properties of cement paste with the aid of a nanoindenter. Besides, this paper also presents a preliminary study on the fatigue behaviour of cement paste at the micrometer level. Miniaturized cantilever beams made of cement paste with different water/cement ratios were statically and cyclically loaded. The micromechanical properties of cement paste were determined based on the measured load-displacement curves. The evolution of fatigue damage was evaluated in terms of the residual displacement, strength, and elastic modulus. The results show that the developed test procedure in this work is able to produce reliable micromechanical properties of cement paste. In addition, little damage was observed in the cantilever beams under the applied stress level of 50% to 70% for 1000 loading cycles. This work may shed some light on studying the fatigue behaviour of concrete in a multiscale manner.

## 1. Introduction

Concrete is the most widely used construction material in the world [1]. As a heterogeneous material, concrete is mainly made of cement, aggregate, and water. It is well acknowledged that the fracture of concrete is a complex phenomenon due to its innate multiphase and multiscale heterogeneity [2]. As a result, the multiscale modelling method is generally adopted to investigate the fracture behaviour of concrete at different length scales [2,3]. By explicitly considering the material structures and material properties of individual constituents at the finer scale, the macroscopic mechanical properties of concrete can be predicted. Therefore, as one of the primary inputs for the multiscale approach, the local mechanical properties of constitutes are needed.

In particular, the cement paste serving as a major component is of great significance with respect to the fracture process of concrete. At the micro level, it is generally recognized that the cement paste includes several different phases: Pores, low density calcium-silicate-hydrate (C-S-H), high density C-S-H, and anhydrous clinker minerals [4,5]. During the last decade, many efforts have been devoted to characterizing the micromechanical properties of cement paste [5,6,7,8,9]. Among them, nanoindentation is a commonly applied method for determination of the local elastic modulus and hardness [5,6]. However, one major concern of this technique is that the local strength of materials is indirectly obtained from the measured hardness, while the correlation between the hardness and strength of cement paste has not been experimentally examined so far [10]. Furthermore, the local mechanical properties of cement paste obtained from nanoindentation tests are largely affected by the spatial heterogeneity of the material [10]. Therefore, the necessity of a more robust test method to determine the micromechanical properties of cement paste is addressed. Recently, Schlangen et al. [11] and Zhang et al. [12] developed a new test procedure to prepare small cement paste cubes (100 µm) with the application of a precision micro-dicing instrument and then to investigate their structural response using a nanoindenter. This promising work successfully provides the experimental evidence for calibration and validation of multiscale modelling techniques. Moreover, to better understand the fracture behaviour of cement paste at the microscale, multiple types of tests should be conducted. Therefore, this concept of small scale testing on cement paste has also been extended to perform splitting tests [13] and three-point bending tests [14]. Similarly, Němeček et al. [9] also conducted small scale bending tests on cantilever micro-beams (20 µm) fabricated by focused ion beam technology and the tensile strength of an individual component in the cement paste was directly measured. However, a major disadvantage of this method is the time-consuming fabrication process of the specimen, which results in a small number of specimens that can be tested. It should be noted that at the microscale, a large scatter of the mechanical properties of cement paste can be expected [15,16]. It means that a sufficient number of tests should be performed to guarantee the statistical reliability [13]. In this paper, the beams with a square cross-section of 380 µm × 380 μm were fabricated by a micro dicing saw. The mechanical properties of cement paste at the microscale were measured by conducting bending tests on miniaturized cantilever beams with the aid of a nanoindenter. The main benefits of this approach are that the mechanical properties can be reliably determined in an easy and straightforward manner. Furthermore, a sufficient number of samples can be tested in a relatively short period of time.

Different from the fracture behaviour under monotonic loading, fatigue fracture of concrete is a process of progressive, permanent internal structural changes, which inevitably result in the changes of the performance of concrete with the elapse of time. This ageing phenomenon of concrete is mainly attributed to the growth of internal microcracks, which eventually coalesce into macrocracks and lead to complete fracture after a sufficient number of cycles [17,18]. Meanwhile, the time-dependent growth of microcracks will accelerate the formation of different gradients in concrete [19,20], and hence, further increase the proneness of concrete to ageing [21]. Although numerous studies have been conducted on the fatigue behaviour of concrete, most of them deal with the global approaches by means of S-N curves or fracture mechanics [22,23,24,25]. The common shortcoming of them is that the inherent heterogeneity of concrete is neglected, and thus the realistic behaviour of concrete under fatigue loading, e.g., the propagation of microcracks, is difficult to be predicted. In order to gain a better understanding of the fatigue fracture of concrete, the multiscale approach is expected to be a proper choice [26,27,28]. According to the study of [27], a mesoscopic model based on the real microstructure was established to investigate the evolution of fatigue damage in concrete, in which the different fatigue damage functions of individual components were assumed. However, the independent fatigue behaviour of different components has never been tested experimentally. To fill this gap, a preliminary study on the fatigue behaviour of cement paste at the micrometre length scale was also carried out in this paper.

## 2. Materials and Methods

### 2.1. Materials and Sample Preparation

In this study, standard grade CEM I 42.5 N Portland cement and deionized water were used to prepare the cement paste. The cement was mixed with water to yield three different water/cement ratios (0.3, 0.4, and 0.5). After mixing, the mixture was cast in plastic cylindrical moulds with a 24 mm diameter and 39 mm height. In order to reduce the amount of entrapped air, the fresh paste was carefully compacted and stirred layer by layer on a vibrating table. Afterwards, the sample was covered with a plastic foil and rotated at a speed of 2.5 rpm at room temperature (20 °C) for 24h, aiming to mitigate the influence of bleeding. All specimens were cured in a sealed condition at room temperature for 28 days. After that, specimens were demoulded and then cut into slices with a thickness of 3 mm. The solvent exchange method was adopted to arrest the hydration reactions of samples by immersing them in isopropanol [29]. A detailed procedure of arresting hydration can be found in [14].

**Figure 1:**
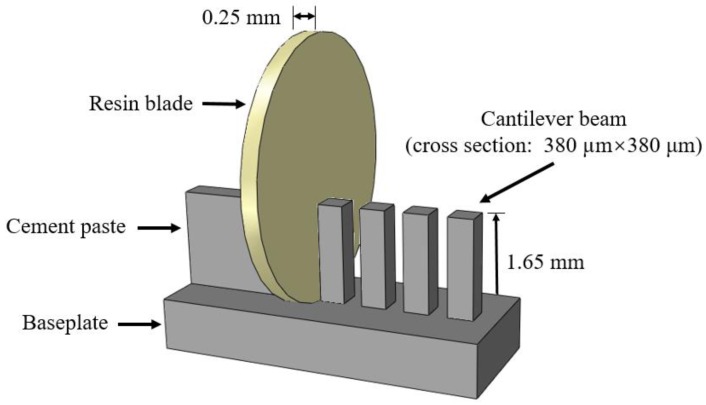
Schematic diagram of sample preparation.

**Figure 2:**
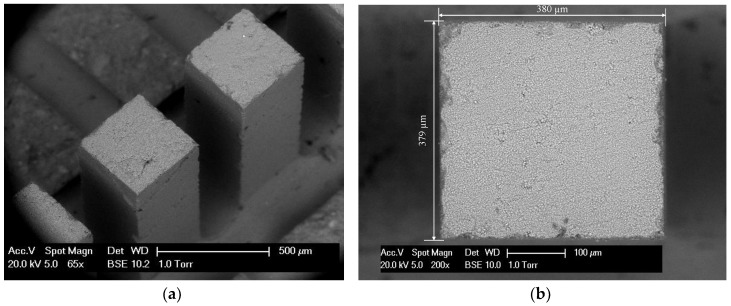
Environmental scanning electron microscope (ESEM) image of: (**a**) A row of cantilever beams; (**b**) cross-section of a cantilever beam.

For the fabrication process of miniaturized cantilever beams, several steps were followed: Firstly, the two ends of slices were further ground using a Struers Labopol-5 thin sectioning machine. To obtain smooth and parallel surfaces, grinding discs with two different grit sizes of 135 μm and 35 µm were used in sequence. The final thickness of the sample was approximately 2.15 mm; the next step is to generate the miniaturized cantilever beams from the thin samples. This is achieved by utilizing a precision micro-dicing machine (MicroAce Series 3 Dicing Saw, Loadpoint Limited, Swindon, UK), which is mainly applied to cut semiconductor wafers. By applying two perpendicular cutting directions and same cutting space, a row of cantilever beams, including at least 20–30 beams, with a square cross section of 380 µm × 380 μm were obtained. The cantilevered length was approximately 1.65 mm and the thickness of the baseplate was less than 500 µm. The cutting process is schematically shown in Figure 1. Afterwards, the cross-section of the beams was examined by using an environmental scanning electron microscope (ESEM). The fabrication process yields an overall accuracy for the cross-sectional dimensions of ±5 μm (Figure 2b).

### 2.2. Static Bending Tests

**Figure 3:**
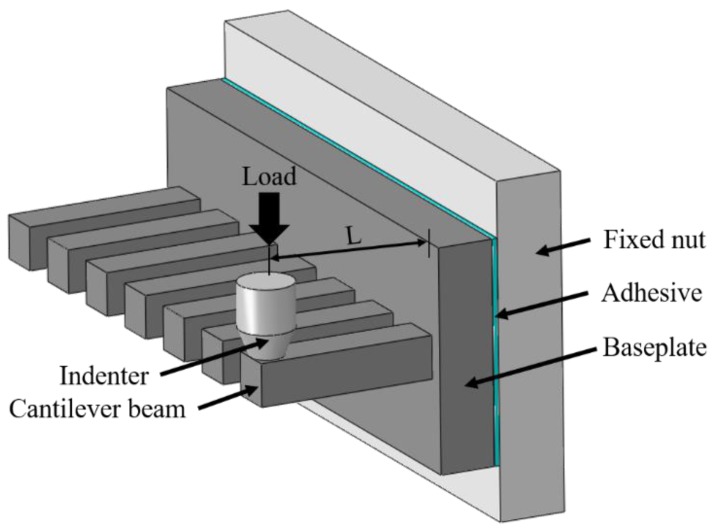
Schematic diagram of test set-up.

For each w/c ratio, a total of 30 cantilever beams were tested under static bending. An Agilent G200 nanoindenter operating with a continuous stiffness measurement (CSM) function was selected to conduct the static bending tests. The CSM technique allows for the continuous measurement of mechanical properties of materials, thus eliminating the need for unloading cycles [30]. In this study, an oscillating force with a harmonic displacement of 2 nm at a frequency of 45 Hz was adopted for the CSM method. The set-up of the static test is illustrated in Figure 3. A nut was first fixed on a metal block, which acts as the supporting base. Then a row of cantilever beams was horizontally attached on the flat wall of the fixed nut using cyanoacrylate adhesive (Loctite Superglue 3). A flat end cylindrical diamond indenter with a diameter of 315 µm was used instead of the mostly used Berkovich indenter (a sharp pyramid tip) for the standard nanoindentation test [11,31,32]. In this way, the possible penetration of the indenter into the specimen was eliminated. The built-in imaging technique of the nanoindenter was used to accurately locate the load point at the central axis of the beams. The load point was always kept around 150 µm from the free end of the beam. The loading procedure was displacement controlled with a 50 nm·s^−1^ loading rate. The cantilever beams were loaded until failure and the load-displacement curves were collected by the nanoindenter. After failure, the distance between the load point and the fracture point on the cantilever beam was measured for the calculation of the stress.

### 2.3. Fatigue Bending Tests

**Figure 4:**
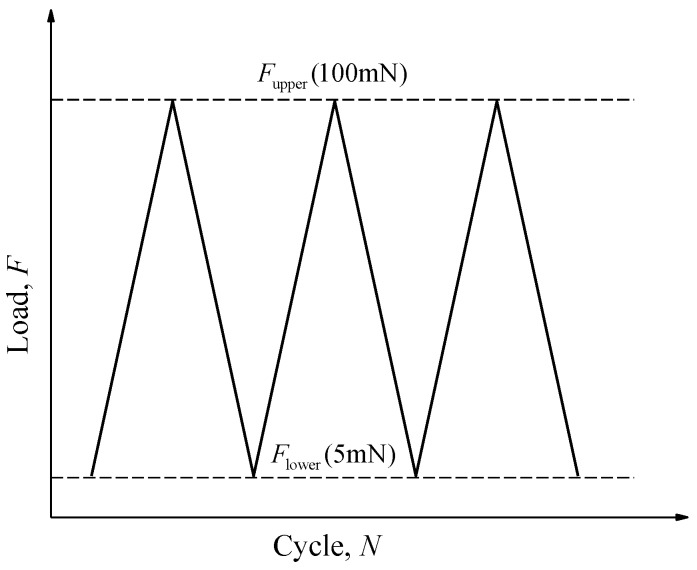
Schematic diagram of the load-cycle curve.

Fatigue tests were performed using the same experimental apparatus (Agilent G200 nanoindenter) as static tests. In total, nine cantilever beams with a w/c ratio of 0.5 were tested under the same upper and lower load. The tests were load-controlled with a constant upper load of 100 mN. The ratio, *R*, between the minimal and maximum load was 0.05. Figure 4 illustrates a schematic diagram of the load-cycle curve used in the fatigue tests. The tests were carried out using the constant amplitude triangular load with a frequency of 0.33 Hz. Note that due to the material heterogeneity, the realistic stress level (tensile stress divided by the static strength) for each specimen is varied even under the same maximum load. For the sake of simplicity, the average value of 63% is adopted to describe the applied stress level. Due to the limitation of test duration in the nanoindenter, four identical load blocks with 250 loading cycles (in total 1000 cycles) were performed on each beam and all of them were conducted in succession without any rest period. Since the fatigue load must be unloaded to zero after completing each load block, the residual displacement was recorded to connect with the succeeding block. Besides, before the end of each load block, the indenter was held for around 50 s at a lower load for the purpose of thermal drift correction [33]. After reaching the target number of cycles, the beams were statically loaded until failure. The load-displacement curves for fatigue tests as well as static tests were recorded by the nanoindenter.

## 3. Results and Discussions

### 3.1. Static Bending Tests

**Figure 5:**
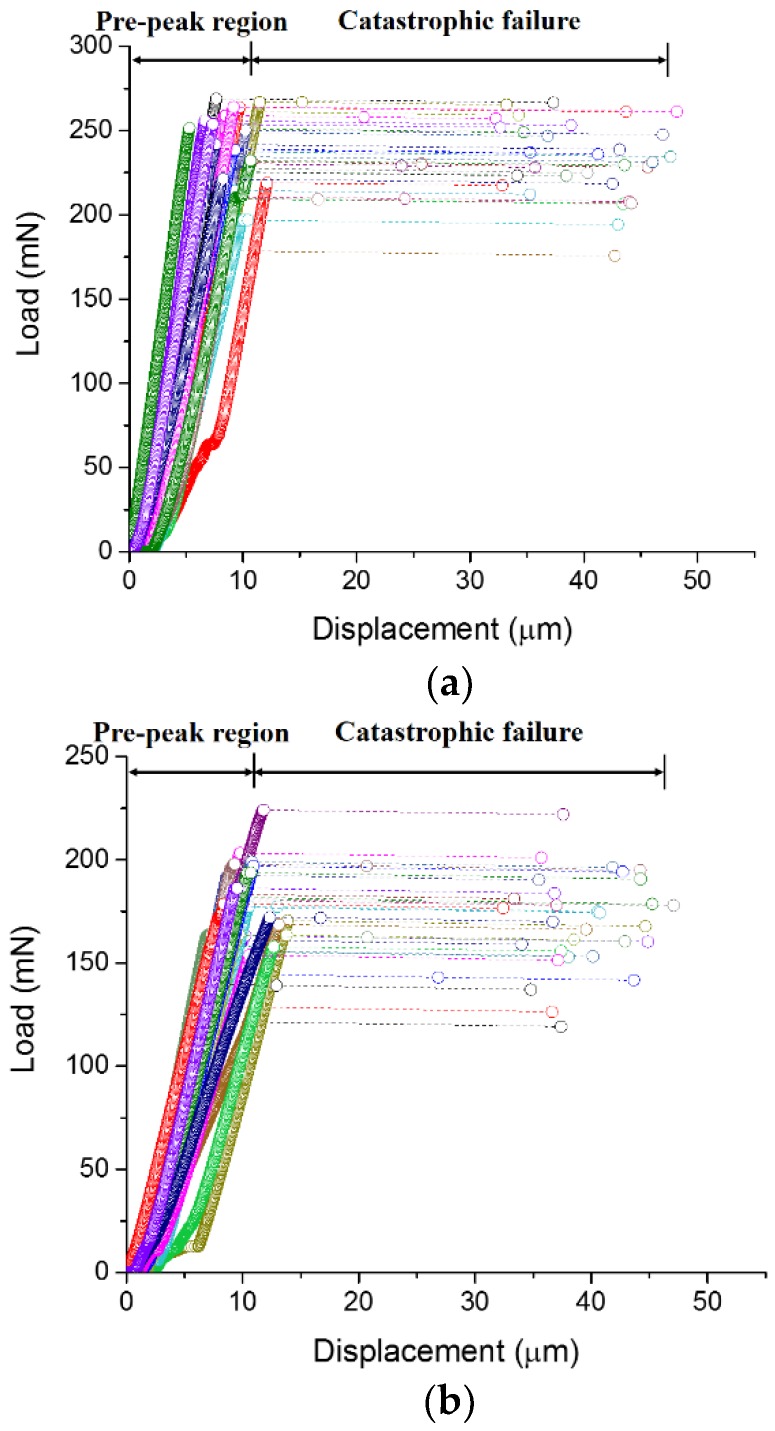

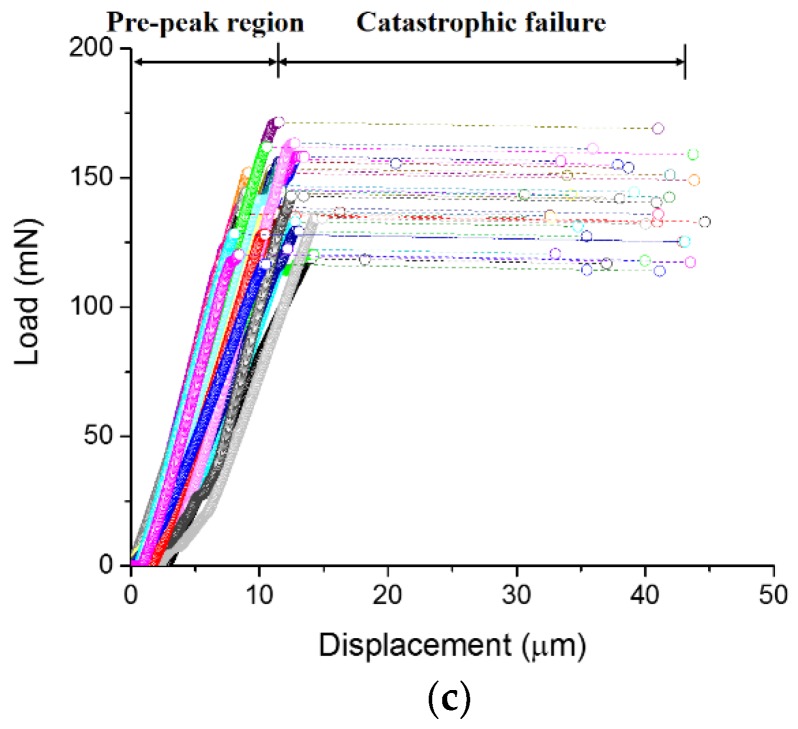
Load-displacement curves of beams with a w/c ratio of (**a**) 0.3; (**b**) 0.4; and (**c**) 0.5.

The load-displacement curves for different w/c ratios are presented in Figure 5. A high degree of repeatability is found for measurements on 30 cantilever beams with the same w/c ratio. It can be seen that the load-displacement curves show two distinct stages. In the first stage, the displacement increases linearly with the increase of load until the maximum load is reached. It is then followed by a rapid burst of displacement in the second stage, where a nearly brittle fracture occurred. Since the displacement control of the nanoindenter is not fast enough to capture the post-peak behaviour of the specimen, it results in an overshoot of the indention tip, thus presenting a horizontal line in the load-displacement curve [34].

**Figure 6:**
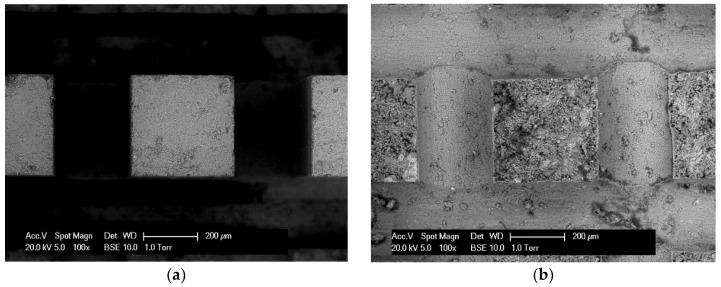
ESEM images of beams (**a**) before fracture; (**b**) after fracture

Figure 6 shows the ESEM images of cantilever beams before and after the bending test. It appears that the tested beam has a rough fracture surface and may be resulted from the randomly distributed defects (pores). It should be noted that the cracks usually initiate at the weakest point near the fixed end of the cantilever beams and eventually lead to the complete fracture.

**Figure 7:**
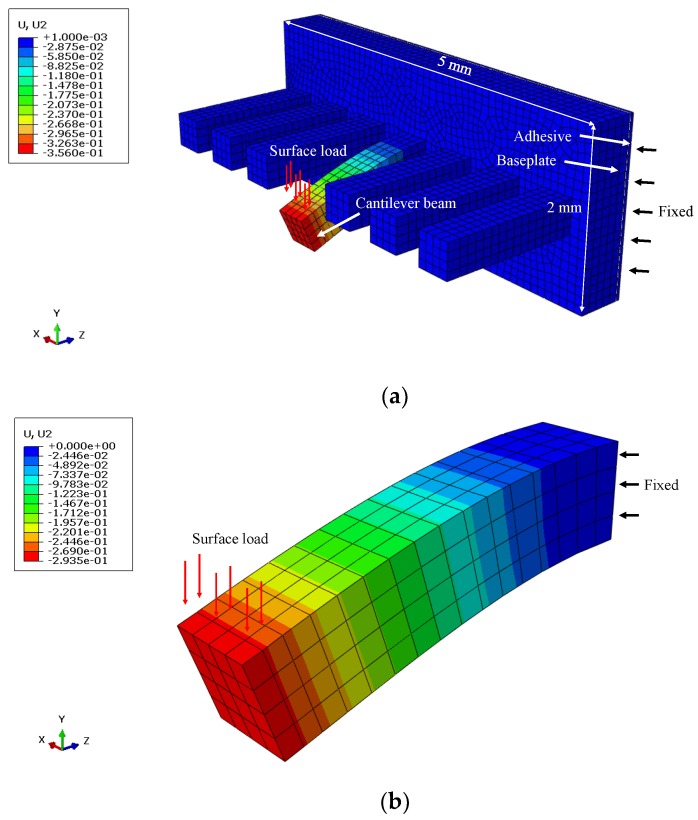
The elastic analysis of (**a**) the cantilever beam with the baseplate and adhesive; (**b**) a single cantilever beam.

The load-displacement curve data is used to estimate the mechanical properties of the cement paste, i.e., the strength and elastic modulus. For static beam experiments, the strength is commonly defined as the tensile stress in the failure plane extreme fiber under maximum load, *F*_max_ [35]. Assuming the beam is loaded without torsion, the strength of beams, *f*_t_, is given by:

(1) $$f_{t}=\frac{F_{\max}d}{I_{y}}\frac{h}{2}$$

where *d* is the distance between the load point and the fracture point, *h* is the side length of the square cross-section, and *I*_y_ = *h*^4^/12 is the moment of inertia. For the calculation of the elastic modulus, *E*, the linear region of the load-displacement curve is used. Note that the effects of shear deformation can be negligible only when the length to depth of the beam is large enough (normally ≥5.0) [9,36,37], which is, however, not the case in this study (around 4.3). Therefore, according to the Timoshenko beam theory [35], the elastic modulus in consideration of the shear effect can be computed as:

(2) $$E=\frac{L^{3}}{3I_{y}}k+\frac{2(1+\nu )L}{\kappa L^{2}}k$$

where *L* is the effective length of the cantilever beam; *k* is the beam stiffness, which is defined as the slope in the linear region of the load-displacement curve; and *ν* and *κ* are the Poisson’s ratio of the cement paste and shear coefficient for rectangular cross-section, respectively. Considering the values reported in the literature [3,7,38,39,40], *ν* = 0.25 and *κ* = 5/6 are used in this study. In addition, it should be borne in mind that the measured load-displacement curves also include the influence of the baseplate and adhesive, which may potentially underestimate the stiffness of the beam [36]. To evaluate the effects of the baseplate and adhesive on the overall stiffness, a finite element model using commercial software, ABAQUS, was established. The model consists of approximately 9000, three dimensional, eight-node brick elements (C3D8R), with a geometry as shown in Figure 3. In this model, the thickness of the adhesive was assumed to be 50 μm, and the elastic moduli of the cement paste and adhesive were set to 15 GPa and 3 GPa, respectively. The thickness of the baseplate used in the model varied from 100 μm to 500 μm. Meanwhile, a single cantilever beam with a fixed end was also simulated for comparison. An example of the numerical model, including the mesh and boundary conditions, is illustrated in Figure 7. The simulation results reveal that the additional deflection caused by the adhesive is not more than 0.5–0.6% of the total beam deflection, which is thought to be negligible. However, the extra deflection caused by the baseplate accounts for around 5–20% of the total beam deflection, largely depending on the thickness of baseplate. For the sake of simplicity, in this study, the extra deflections caused by the adhesive and baseplate are not taken into account in the estimate of the elastic modulus. Nevertheless, continuous improvement of the test procedure is required to further minimize the discrepancies in measured material properties, which is not attributed to the materials themselves.

The calculated mean value and standard deviation of the strength and elastic modulus are summarized in Table 1, together with the data reported in the literature [14] (in brackets). As expected, both the strength and elastic modulus decrease with an increasing w/c ratio. It can also be seen that the calculated elastic modulus agrees well with the literature results mentioned by [14], while the calculated strength is almost 1.5–2.0 times higher than the results in the literature. It is believed that the longer beam (i.e., 12 mm) used in [14] is the major reason for the discrepancy in the calculated strength despite the possible influence of different boundary conditions. The observed decrease in strength for larger samples is a common feature for quasi-brittle material and can be explained by the size effect [41,42,43].

### 3.2. Fatigue Bending Tests

#### 3.2.1. Load-Displacement Curve

**Figure 8:**
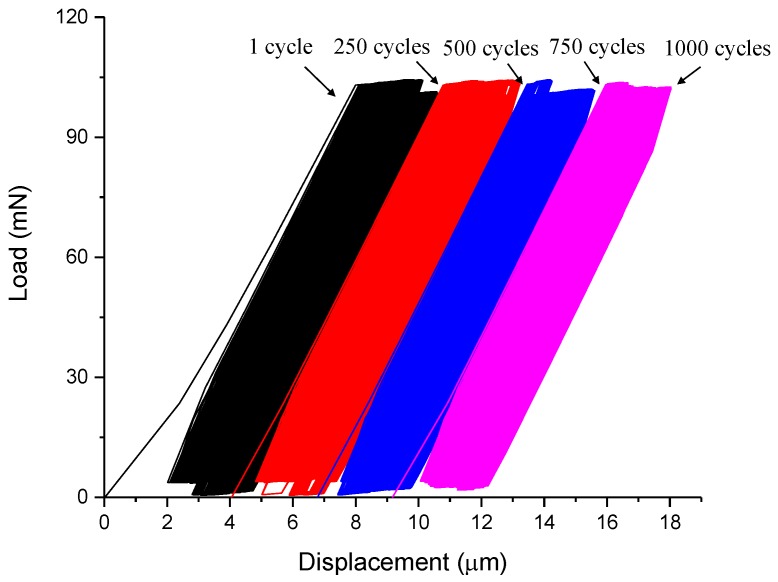
A typical load-displacement curve for the fatigue test.

**Figure 9:**
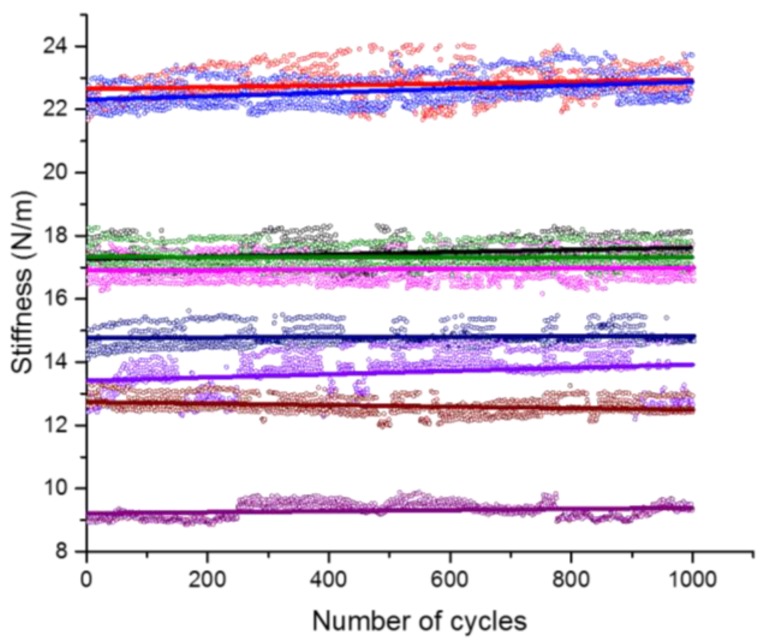
The loading stiffness versus the number of cycles for all fatigue tests (different colors represent different specimens).

The typical measured load-displacement curve of the fatigue test is shown in Figure 8. The figure shows that the load-displacement relation for each cycle is approximately linear, and also the residual displacement are accumulated with the increase of loading cycles. The variation of stiffness during loading characterized by the slope of the loading curve for all fatigue tests are plotted in Figure 9, together with the line of best fit. It is found that the loading stiffness changes very little with the number of cycles, which is associated with a slow accumulation of damage during the fatigue loading. It should be noted that the fatigue response of material is very sensitive to the flaws and defects [44,45,46]. Consequently, when compared with the mortar or concrete at the larger scale, the cement paste at the micro scale exhibits higher fatigue resistance due to less initial defects, such as the porous interfacial transition zone (ITZ) [47]. 

**Figure 10:**
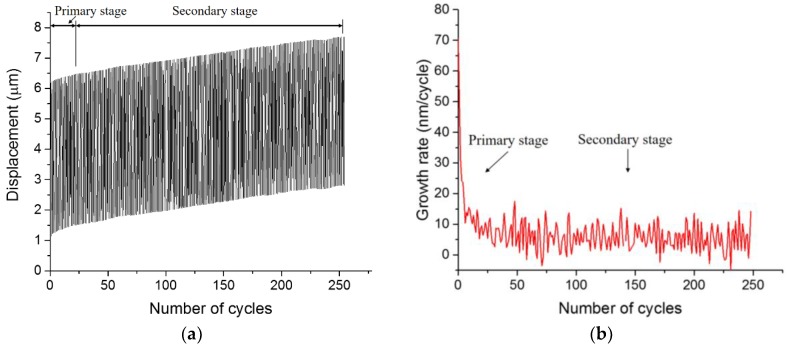
A typical curve of (**a**) displacement versus the number of cycles; (**b**) growth rate of displacement with cycles.

Although no evident decline of loading stiffness can be identified in all fatigue tests, the relation between the displacement and number of cycles exhibits a typical fatigue damage evolution curve [18,48,49,50], see Figure 10a. The increasing displacement under each loading block can be divided into a transient primary stage and a steady secondary stage. It can also be seen from Figure 10b that the growth rate of displacement decreases rapidly in the primary stage and reaches a constant value, with relatively small fluctuations in the secondary stage. These two stages are mainly related to the formation and progressive growth of internal microcracks in cementitious materials, respectively [24,51].

#### 3.2.2. Growth Rate of Residual Displacement

**Figure 11:**
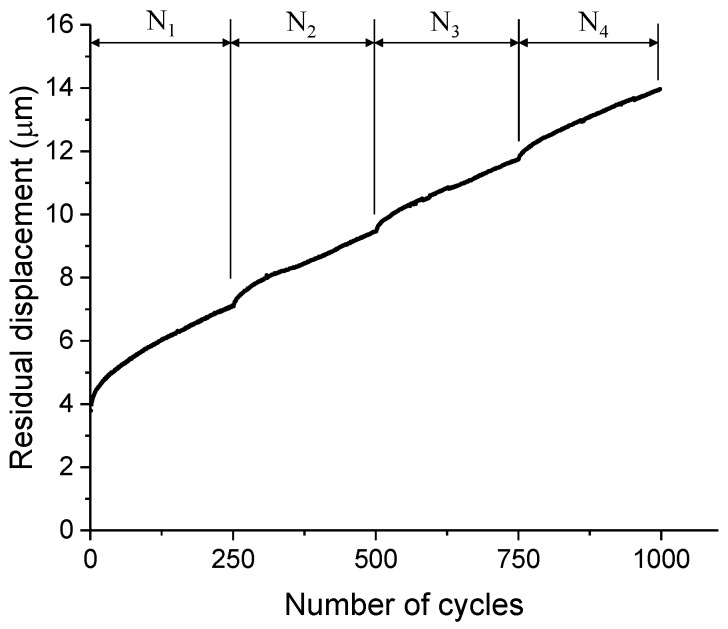
A typical curve for the development of residual displacement (N_1_, N_2_, N_3_, and N_4_ denote the four load blocks).

**Figure 12:**
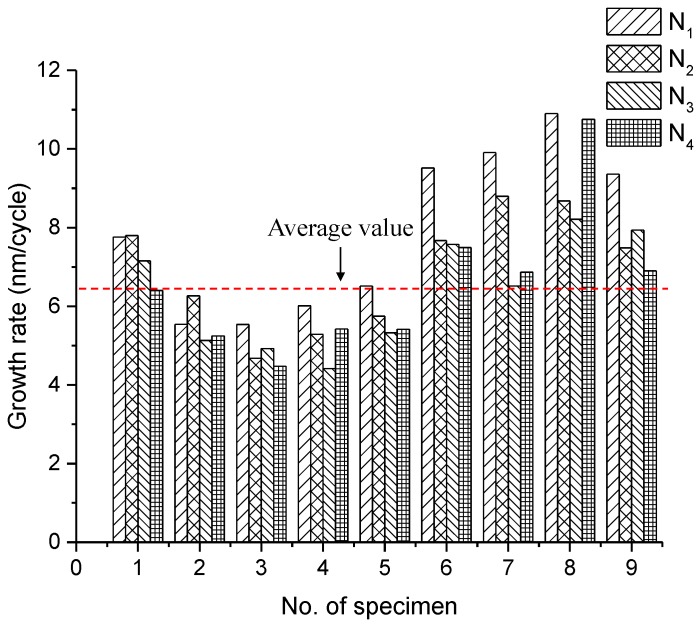
The growth rate of residual displacement for all specimens (N_1_, N_2_, N_3_, and N_4_ denote the four load blocks).

As an important indicator of the fatigue damage, the residual displacements at a lower load are extracted from the load-displacement curve. A typical curve for the development of residual displacement under four identical loading blocks is plotted in Figure 11. Despite the discontinuity between each load block, the development of residual displacement shows an overall increasing trend with nearly the same slope. This indicates a stable propagation of microcracks in cantilever beams even with the interruption of cyclic loading. Figure 12 presents the constant growth rate of the residual displacement for each load block as well as the average value of all tests (6.47 ± 1.01 nm/cycle). Note that the data with large deviations are excluded in the calculation of the average value. Figure 12 indicates that the growth rate of each load block shows a high degree of consistency. Nevertheless, the value between each specimen is relatively scattered, mainly due to the natural heterogeneity of cementitious materials.

#### 3.2.3. Residual Mechanical Properties

**Figure 13:**
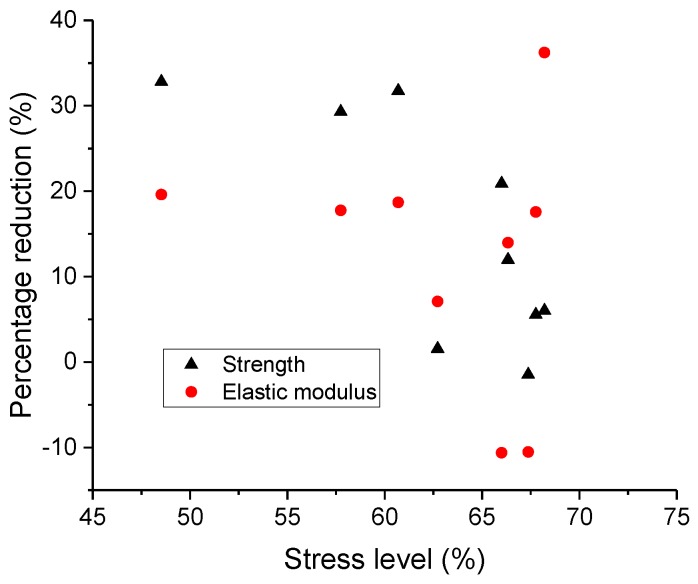
The relation between the percentage reduction of mechanical properties and the stress level.

To quantitatively assess the degree of fatigue damage, all damaged cantilever beams were loaded statically to failure. The percentage reduction of the strength and elastic modulus for each beam were plotted against the stress level in Figure 13. The obtained average percentage reduction of the strength and elastic modulus are 15.29% and 15.78%, respectively.

**Figure 14:**
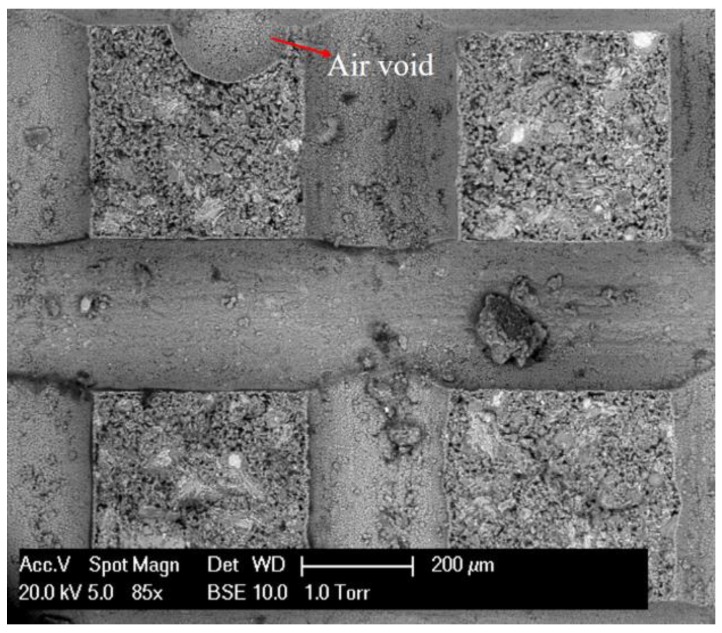
The ESEM image of a large air void detected in the fractured cross-section of cantilever beams.

As expected, a large scatter can be observed in this diagram. For instance, the percentage reduction of strength ranges from 1% to 20% even under the same stress level of 67%, while the percentage reduction of the elastic modulus lies in a wider range of −10% to 36%. A downtrend inferred from Figure 13 indicates that the higher stress level leads to the lower percentage reduction of mechanical properties, which seems to be unrealistic. On the other hand, since the number of tests is relatively small, it is not enough to make a rational judgement of the results. Nevertheless, the observed scatter in Figure 13 implies that the applied stress level may not be a proper parameter to correlate the residual mechanical properties at this length scale. The cause of the failed interpretation of the results is mainly attributed to the assumption of a constant strength. However, cement paste at the micro scale contains different size of pores and initial defects [5,7,52]. Most of them are formed during the hydration process [53] or induced by the external factors, such as casting and vibration. For instance, a large air void can be detected in the cross-section of the cantilever beam, as shown in Figure 14. Therefore, the randomly distributed pores may result in the difference of original strength. This is consistent with previous numerical work [52], in which the predicted mechanical properties of cement paste at the microscale were largely influenced by the real microstructures.

**Figure 15:**
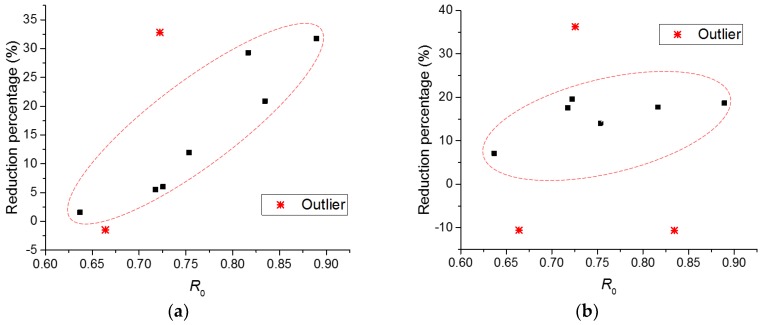
The relation between the percentage reductions of (**a**) strength; (**b**) elastic modulus against the ratio, *R*_0_.

To consider the individual variation of strength of each specimen, the ratio of applied stress to residual strength (denoted as *R*_0_) is adopted to evaluate the fatigue damage. The relation between the residual mechanical properties and the ratio, *R*_0_, are shown in Figure 15. A clear trend can be found that the percentage reduction of strength and elastic modulus increase with the increase of the ratio, *R*_0_. It indicates that the employed parameter is likely to give a proper analysis of the data, but this only applies to the beams undergoing low fatigue damage. It should also be kept in mind that to acquire a reliable prediction of fatigue behaviour of the cement paste, a large number of tests is needed.

## 4. Conclusions

In this study, the nanoindenter technique was used to investigate the micromechanical properties of cement paste. This work also represents the first attempt in studying the fatigue behaviour of cement paste at the micrometer length scale. From the results of the current study, we can conclude that the employed test procedure is able to obtain reliable mechanical properties of cement paste at the micro scale. In addition, the cement paste at the microscale exhibits higher fatigue resistance due to less initial damage. The fatigue damage evolution can be quantitatively assessed in terms of the residual displacement and the residual mechanical properties. In consideration of the heterogeneous microstructure of cement paste, the ratio of stress to residual strength is suggested to properly analyze the evolution of fatigue damage. This study provides new insights into the fatigue behaviour of cement paste, which may shed some light for future studies on the multiscale modelling of the ageing process of concrete subjected to fatigue loading. Systematic and additional studies are required to test the effect of the w/c ratio, loading history, and rest period on the fatigue behaviour of cement paste at the microscale. More importantly, the fatigue behaviour of ITZ at this scale needs to be further investigated.

## Figures and Tables

**Table 1:** Calculated mechanical properties, compared with previous work [14].

w/c Ratio	Strength (MPa)	Elastic Modulus (GPa)
0.3	31.34 ± 3.70 (20.28) ^1^	18.85 ± 3.23 (16.68) ^1^
0.4	25.27 ± 3.23 (15.31) ^1^	13.97 ± 1.98 (12.79) ^1^
0.5	22.37 ± 2.31 (11.71) ^1^	10.80 ± 2.44 (9.09) ^1^

^1^ The value in brackets are from the reference [14].
